# Evolution Characters and Influencing Factors of Regional Eco-Efficiency in a Developing Country: Evidence from Mongolia

**DOI:** 10.3390/ijerph182010719

**Published:** 2021-10-13

**Authors:** Bing Xia, Suocheng Dong, Yu Li, Zehong Li, Dongqi Sun, Wenbiao Zhang, Wenlong Li

**Affiliations:** 1 Institute of Geographic Sciences and Natural Resources Research, Chinese Academy of Sciences, Beijing 100101, China; xiab.16b@igsnrr.ac.cn (B.X.); liy@igsnrr.ac.cn (Y.L.); lizehong@igsnrr.ac.cn (Z.L.); sundq@igsnrr.ac.cn (D.S.); zhangwenbiaozwb@163.com (W.Z.); nmgliwenlong@126.com (W.L.); 2 College of Resources and Environment, University of Chinese Academy of Sciences, Beijing 100049, China; 3 Resources and Environment Economy College, Inner Mongolia University of Finance and Economics, Huhhot 010070, China

**Keywords:** eco-efficiency, SBM model, evolution characters, influencing factors, Mongolia, developing countries

## Abstract

The sandstorm in 2021 in East Asia demonstrated the ecological issues that culminated for decades in Mongolia. Mongolia is facing challenges to realize green and sustainable development. This article aims to increase the understanding of eco-efficiency and its influencing factors in Mongolia and to provide a reference for similar developing countries and regions to achieve green and sustainable development. This article used the Slacks-Based Measure of Efficiency (SBM) model with advantages of dimension freedom and unit variable to estimate the economic efficiency and eco-efficiency of 22 provinces in Mongolia from 2007 to 2016; energy consumption and undesirable environmental outputs were taken as ecological/environmental indicators in the input and output system of regional eco-efficiency in Mongolia, combining traditional indicators of economic efficiency to build Mongolia’s eco-efficiency input–output framework. This article applied hot spot analysis and gravity center analysis to reveal the temporal and spatial evolution characters of eco-efficiency in Mongolia. Finally, the article applied panel Tobit regression to analyze the influencing factors of eco-efficiency. We were found that Mongolia’s eco-efficiency slightly improved from 0.7379 in 2007 to 0.7673 in 2016, lower than the economic efficiency. The high eco-efficiency provinces appeared in the capital Ulaanbaatar and its surrounding areas, showing an obvious spatial spillover effect. The low eco-efficiency provinces were mainly in the undeveloped western region. The relationship between per capita GDP and eco-efficiency was U-shaped and consistent with environmental Kuznets theory. Accelerating economic growth, optimizing population distribution, and improving energy structure and green technology can improve Mongolia’s eco-efficiency.

## 1. Introduction

With the background of global climate change, destruction of the ecological environment, and the competition between humans and the land, in 2015, the United Nations launched the sustainable development goals (SDGs) [1], which has opened a new stage of global sustainable development [2]. Additionally, the coordinated development of the economy and ecological environment deserved more consideration of global sustainable development. However, in developing countries, environment and natural resource management have apparently not yet been given high priority [3], with an increasing number of case studies from developing countries reporting the failure of sustainable development to achieve ideal synergies between the environment, economy, and society [4]. In fact, for developing countries, economic development and poverty eradication are fundamental to sustainable development [5]. Therefore, for developing countries with a fragile ecology and more vulnerable to climate change, with urgent needs regarding economic and social development, how to protect the ecological environment is a significant issue to achieving the global SDGs.

Mongolia is located in the Mongolian Plateau, with less than 1% arable land [6]. Additionally, only 7.85% of areas are actually covered by forests; most of the land is covered by discontinuous permafrost, which makes construction, road-building, and mining difficult [7]. Moreover, Mongolia is mainly typical drylands, which are an essential component of the Earth System and are among the most vulnerable to climate change [8]. Nearly 77% of land areas have seen changes in vegetation cover, plant species, and biomass production, and soil erosion from wind and water run-off is posing threats to ecosystems [9]. The ecological environment of Mongolia is fragile, and has an important influence on the ecological environment of East and Central Asia. In recent years, the problem of air pollution along the north–south railway in Mongolia, especially in Ulaanbaatar, Joel, Darhan and other cities, has become increasingly serious, and the pressure on the ecological environment is increasing day by day [10]. Between mid-March and mid-April 2021, the country encountered violent gusts of wind, the Mongolia cyclone, and the largest sandstorms in a decade, causing extensive damages nationwide and trans-regional impact in East Asia, including northern China, Japan, and most parts of South Korea, and the ecological issues have been culminated for decades in Mongolia [11]. On the other hand, as the largest landlocked country in East Asia, Mongolia’s economic development is relatively backward. According to the statistics of the World Bank, Mongolia’s per capita GDP in 2019 was USD 4340, less than half of the USD 10,216 of China and USD 11,498 of Russia [12], the main neighboring countries. Economic development and poverty eradication are the objective requirements of human development, which is important for developing countries. The government of Mongolia has attempted to achieve green and sustainable development in economics. In 2016, the government adopted an Action Plan for the Implementation of the Green Development Policy for the period 2016–2030 [13]. However, according to the United Nations report of the Sustainability Outlook of Mongolia, it still had significant gaps in the economic and ecological environment to achieve the SDGs, especially regarding goal 7 of clean, renewable energy and goal 8 of growth of GDP [9]. Therefore, facing the fragile ecological environment and gaps to the SDGs, economic and social development, considering the influence of the ecological environment, have been the realistic needs and national strategy for Mongolia.

Eco-efficiency was first proposed by the World Business Council for Sustainable Development (WBCSD), wherein eco-efficiency is achieved by the delivery of competitively priced goods and services that satisfy human needs and raise the quality of life while progressively reducing ecological impacts and resource intensity throughout the life cycle to a level at least in line with the Earth’s estimated carrying capacity [14]. It is an indicator that can evaluate both economic development and influence on the ecological environment [15]. Regional eco-efficiency is a scientific tool to measure the economic development of a region under the constraints of the ecological environment [16,17,18]. Its purpose is to achieve more economic output with a lesser ecological/environmental impact and emphasize the optimization between ecological/environmental impact and economic value [19,20,21,22]. Regional eco-efficiency is of great significance to achieve regional economic growth, alleviate energy shortage, and reduce environmental pollution; therefore, regional eco-efficiency is relevant to SDGs of economic growth and affordable and clean energy [23,24] (Goal 8, 7). With the background of the uncertain global situation with a more severe competition between humans and land after COVID-19 [25], regional eco-efficiency analysis could become an important scientific tool to evaluate both the ecological/environmental and economic effects for one region or country to achieve the SDGs of economic growth and affordable and clean energy.

Regional eco-efficiency has grown up to be a hot topic in the research of green sustainable development in recent years. The study of regional eco-efficiency has been carried out at national, provincial, and city scales. Data envelopment analysis (DEA) is the most widely used research method for measuring regional eco-efficiency, and scholars further analyze regional eco-efficiency by combining spatial evolution and influencing factors. On the national scale, Camioto et al. (2016) analyzed the energy efficiency of G7 (Canada, France, Germany, Italy, Japan, the United Kingdom, and the United States) and the BRICS countries (Brazil, Russia, India, China, and South Africa) under the total factor structure with the Slacks-Based Measure model(SBM), and found that the energy efficiency of G7 countries was above 95%; the social conditions such as longevity and income distribution were the main factors to improve the energy efficiency of G7 countries, while the investment of low energy technology was the main influencing factor on the energy efficiency of BRIC countries [26]. Moutinho et al. (2021) analyzed the regional eco-efficiency of the 27 European countries from 2008 to 2018 using the two-stage DEA model and found that the CO_2_/area and CH_4_/area decreased the eco-efficiency score [27]. On the provincial scale, Yang et al. (2020) applied the directional–distance–function model (DDF) to estimate the regional eco-efficiency in China during 2008–2017 and found that promoting production performance was crucial to improving the eco-efficiency for sustainable development in China [19]. Ren et al. (2020) applied the network DEA model to measure regional eco-efficiency in China and found that China’s overall eco-efficiency was still at a low level, and economic growth, marketization, and social input were the main influencing factors [18]. Huang et al. (2018) analyzed the regional eco-efficiency of 30 provinces and regions of China in the period 2001–2014, based on the economic efficiency, energy efficiency, and environmental efficiency, with a Meta-US-SBM model, and found that the different provinces and regions showed significant spatial heterogeneity in different development modes [28]. On the city scale, Ren et al. (2020) applied the Hybrid distance model (S-EBM) to measure the eco-efficiency of 283 prefecture-level cities in China from 2003 to 2013 and found that an increase in eco-efficiency in surrounding cities would improve eco-efficiency in local cities [29]. Zhang et al. (2020) analyzed the regional eco-efficiency of cities along the lower reaches of the Yellow River from 2007 to 2018 using the super SBM model and found that affluence and technological progress were the main drivers of eco-efficiency [30]. Yao et al. (2021) used the epsilon-based measure data envelopment analysis (EBM-DEA) model to analyze the regional eco-efficiency of 28 Russian cities along the Northern Sea Route and found that the cities with higher levels of GRPs per capita had higher eco-efficiency [31].

Existing research has been carried out from different research scales, built a consistent input–output system of eco-efficiency, and analyzed the influencing factors of eco-efficiency from the investment level, green technology, energy tax, local policies, and national economic development. The research objects of regional eco-efficiency mainly concentrated in the European Union, G7, BRICS, especially focused on China. However, few studies were performed on developing countries and regions. Moutinho et al. (2021), using a log-linear Translog production function, analyzed eco-efficiency in Asian and African countries and found that labor and renewable energy share increase eco-efficiency in these countries [23]. Xue et al. (2020) evaluated the urban eco-efficiency in Western China by the undesirable-super-slack-based measure (US- SBM) and found there was a spatial spill-over impact of urban eco-efficiency in Western China, and economic development and urbanization have a “U” shaped impact on Western China’s urban eco-efficiency [32]. These types of countries or regions often need to pay more attention to the development of ecology and the economy to achieve win–win sustainable development of the eco-economy. Mongolia, as a typical developing country with a fragile ecological environment, has attracted a few studies on the relationship between economic and ecological environments. Khalid et al. (2014) analyzed the relationship between CO_2_ emission and economic growth, energy consumption, and trade openness in Mongolia and confirmed the existence of the Environmental Kuznets Curve for CO_2_ emission in Mongolia [33]. Guo et al. (2020) applied multiregional input–output analysis to calculate the embodied greenhouse gas emissions in Mongolia and found that household consumption and gross fixed capital formation were the driving factors for the increase in embodied GHG emissions [34]. The existing studies are valuable to find the relationship between ecological/environmental impacts and economic development in Mongolia; however, the research on eco-efficiency evaluating economic growth and ecological/environmental protection is still insufficient. The research on regional eco-efficiency can provide scientific guidance for developing countries (for example, Mongolia) with a fragile ecological environment to achieve the SDGs of economic growth and affordable and clean energy.

Therefore, this study introduced regional eco-efficiency as a tool to measure both economic development and ecological environment impacts in Mongolia. We analyzed the eco-efficiency spatial pattern and evolution characters of the 22 provinces of Mongolia, then analyzed the influencing factors by econometric analysis. This work would like to provide a framework of regional eco-efficiency analysis from “estimate” to “evolution” to “coupling” to “drivers” and provide a scientific basis and theoretical support for sustainable development policies and achieve goals of economic growth and ecological/environmentally sustainable development in developing countries or regions, especially those with a fragile ecological environment—for example, Mongolia, Pakistan, and the Northwest of China. This work could be useful to promote the above developing countries to achieve green and sustainable development and SDGs of economic growth and affordable and clean energy.

## 2. Materials and Methods

**Figure 1:**
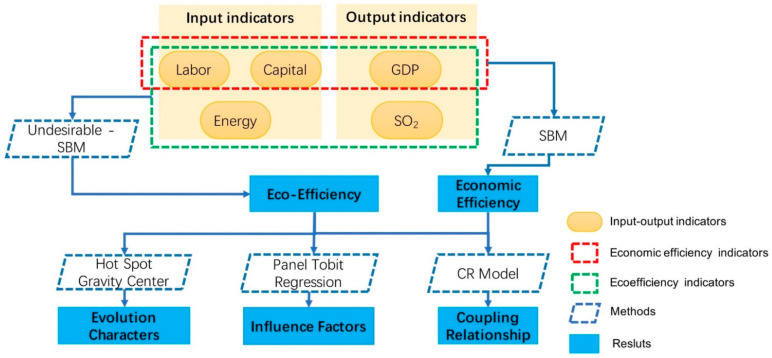
Research framework.

This article applied the Slacks-Based Measure (SBM) model to evaluate economic efficiency and applied Undesirable SBM to evaluate eco-efficiency in Mongolia. Then, we used Hot Spot and Gravity Center model to analyze the temporal and spatial evolution characters and used panel Tobit regression to analyze the influencing factors of eco-efficiency. At last, we applied the Coupling Relationship (CR) model to analyze the coupling relationship between economic efficiency and eco-efficiency in Mongolia (Figure 1).

### 2.1. The Analysis Method of Economic Efficiency and Eco-Efficiency: SBM Model

The data envelopment analysis (DEA) method has some advantages in the application of evaluating eco-efficiency [35,36,37]. The model is without the unified index unit, without clearing the function relation, without estimating the parameters. Further, the undesired output indicator can also be included, and the input–output indicator added is flexible in selection. There are many extended models of DEA, and the main DEA models widely used to evaluate eco-efficiency include traditional CCR and BCC models [38,39], SBM model with relaxed variables in objective function [40,41,42], DDF model of directional distance function [43,44], and Network DEA model [45,46,47,48]. This study used the Slacks-Based Measure of Efficiency (SBM) model with advantages of being dimension-free and unit-variable [49,50,51] to estimate the efficiency and eco-efficiency in Mongolia. In this study, we took 22 provinces of Mongolia as research space units and estimated the economic efficiency and eco-efficiency of 22 provinces contemporaneous (each year) [52].

The economic efficiency analysis of each spatial unit includes input and output vectors, expressed as $x\in R^{m}$, $y\in R^{s}$, to define the matrix *X*, *Y* as follows: $\left[X\right]={\left[x_{1},\cdots ,x_{n}\right]}^{T}\in R^{m\times n}$, $\left[Y\right]={\left[y_{1},\cdots ,y_{n}\right]}^{T}\in R^{s\times n}$, *X* > 0, *Y* > 0. Production possibility set *P* is $\left\{\left(x,y\right)|x\geq \lambda x,y\leq \lambda Y,\lambda \geq 0\right\}$. The basic expression of SBM is as follows [53]:

(1) $$\begin{array}{c} P^{*}=min\frac{1-\frac{1}{m}\sum_{i=1}^{m}\frac{s_{i}^{-}}{x_{i0}}}{1+\frac{1}{s}\sum_{r=1}^{s}\frac{s_{r}^{+}}{y_{r0}}}\\ \;\mathrm{St}.\;x_{0}=X\lambda +s^{-}\\ \;y_{0}=Y\lambda -s^{+}\\ \lambda \geq 0,s^{-}\geq 0,s^{+}\geq 0 \end{array}$$

where *s* represents the slack variable of input and output, and λ is the weight vector. The objective function $P^{*}$ is strictly based on *s^−^*, *s^+^*, and $0\leq P^{*}\leq 1$. If $x_{i0}$ = 0, then we delete the term $s_{i}^{-}$/$x_{i0}$ in the objective function. If $y_{r0}$ < 0, then we replace it with a very small positive number so that the term $s_{r}^{+}$/$y_{r0}$ plays a role of penalty.

This article applied Undesirable SBM model with undesirable outputs to measure the eco-efficiency of Mongolia. Each space unit includes three vectors: input, expected output and undesirable output, expressed as $x\in R^{m}$, $y^{g}\in R^{s_{1}}$, $y^{b}\in R^{s_{2}}$, to define matrices *X, Y^g^, Y^b^* as follows: $\left[X\right]={\left[x_{1},\cdots ,x_{n}\right]}^{T}\in R^{m\times n}$, $\left[Y^{g}\right]={\left[y_{1}^{g},\cdots ,y_{n}^{g}\right]}^{T}\in R^{s_{1}\times n}$ and $\left[Y^{b}\right]={\left[y_{1}^{b},\cdots ,y_{n}^{b}\right]}^{T}\in R^{s_{2}\times n}$, *X* > 0, *Y^g^* > 0, *Y^b^* > 0. The Undesirable SBM model based on variable returns to scale expressed as follows [54]:

(2) $$\begin{array}{c} P^{*}=min\frac{1-\frac{1}{m}\sum_{i=1}^{m}\frac{s_{i}^{-}}{x_{i0}}}{1+\frac{1}{s_{1}+s_{2}}\left[\sum_{r=1}^{s_{1}}\frac{s_{r}^{g}}{y_{r0}^{g}}+\sum_{r=1}^{s_{2}}\frac{s_{r}^{b}}{y_{r0}^{b}}\right]}\\ \mathrm{St}.\;x_{0}=X\lambda +s^{-}\\ y_{0}=Y\lambda -s^{+}\\ \lambda \geq 0,s^{-}\geq 0,s^{+}\geq 0 \end{array}$$

where *s* represents the slack variable of input and output, and λ is the weight vector. The objective function $P^{*}$ is strictly based on *s^−^*, *s^g^*, *s^b^*, and $0\leq P^{*}\leq 1$. For a specific space unit, when and only when $P^{*}=1$, and *s^−^*, *s^g^*, *s^b^* are all 0, eco-efficiency is at the optimum front. Conversely, it shows that the eco-efficiency of the spatial unit is ineffective, and there is a necessity to improve the input–output variables. Mongolia’s eco-efficiency value in this study (abbreviated as *EE*) was divided into five levels. 0.00 < *EE* < 0.40 was a province of inefficiency, 0.41 < *EE* < 0.60 was a low-efficiency province, 0.61 < *EE* < 0.80 was a province to be improved, 0.81 < *EE* < 0.99 was an efficient province, and *EE* = 1 is a highly efficient province. This article applied DEA_Solver5.0 software for SBM model and Undesirable SBM model.

### 2.2. Analysis Method of Spatial Pattern: Analysis of Hot Spot and Center of Gravity

The Getis-Ord G_i_* index is put forward by Getis and Ord, and we can find subregions with different attribute values in the study area. This can be expressed as follows [55]:

(3) $$G_{i}^{*}=\frac{\sum_{j=1}^{n}\varphi_{i,j}x_{j}-(\frac{1}{n}\sum_{j=1}^{n}x_{j})\times \sum_{j=1}^{n}\varphi_{i,j}}{\sqrt{\frac{\sum_{j=1}^{n}x_{j}}{n}-{\left(\frac{1}{n}\sum_{j=1}^{n}x_{j}\right)}^{2}}\times \sqrt{\frac{n\sum_{j=1}^{n}\varphi_{i,j}^{2}-{\left(\sum_{j=1}^{n}\varphi_{i,j}\right)}^{2}}{n-1}}}$$

where *x_j_* is the attribute value of factor *j*, *φ_i,j_* is the spatial weight between elements *i* and *j*, and *n* is the total number of elements. According to the G_i_* index, we can obtain the provinces with high attribute value (hot spot) or low attribute value (cold spot). The Getis-Ord G_i_* index was divided into 7 types. Cold spot provinces had negative correlation saliency of 0.01; sub cold spot provinces had negative correlation of 0.05, sub-sub cold spot provinces had negative correlation of 0.1. While hot spot provinces had a positive correlation in 0.01, sub hot spot provinces had positive correlation of 0.05, sub-sub hot spot provinces had positive correlation of 0.1. Additionally, in the remaining provinces, the correlation was not significant. The hot spot and cold spot of eco-efficiency in each province of Mongolia can represent high-concentration area and low-concentration area of eco-efficiency, respectively.

The center of gravity coordinates, originally derived from geometric analysis in mathematics, is a measure of the spatial characteristics of an object, usually used on objects with geometric coordinates. The center of gravity coordinates in geography can analyze the change of the spatial center of a certain attribute value, which can express the clustering characteristics and moving trajectories of spatial attributes. Its mathematical expression is as follows [56]:

(4) $$p_{t}\left(x_{i},y_{i}\right)=\left[\frac{\sum_{i=1}^{n}ee_{i}x_{i}}{\sum_{i=1}^{n}ee_{i}},\frac{\sum_{i=1}^{n}ee_{i}y_{i}}{\sum_{i=1}^{n}ee_{i}}\right]$$

where *p_t_*(*x_i_*,*y_i_*) represents the center of gravity coordinates of Mongolia’s eco-efficiency in *t* year; *ee_i_* represents the eco-efficiency value of the *i* province; *x_i_*, and *y_i_* represents the center of coordinates of *i* province. This article applied ArcGIS10.4 software to evaluate Getis-Ord G_i_* index and center of gravity.

### 2.3. Influencing Factors Analysis Method: Panel Tobit Analysis

The Tobit regression model is proposed by Tobin [57]. It belongs to the regression model with limited dependent variables. It can solve the problem of modeling restricted or truncated dependent variables [58]. The Tobit model has been widely used to investigate the influencing factors of eco-efficiency. Because eco-efficiency evaluated by Undesirable SBM always has a value from 0 to 1, it is not suitable to use ordinary least squares (OLS) for coefficient estimation [59]. Moreover, the Tobit regression model with fixed effects is difficult to use to perform conditional maximum likelihood estimation, such as in the Logit or Counting model with fixed effects, due to the inadequate statistics of individual heterogeneity. Therefore, we selected panel random effect Tobit regression model to identify the influencing factors of regional eco-efficiency in Mongolia. The model expression is as follows [55]:

(5) $$y_{it}^{*}=\alpha x_{it}+\varepsilon_{it}\;y_{it}=\left\{\begin{array}{c} y_{it}^{*},\;y_{it}^{*}\geq 0\;\\ 0,\;y_{it}^{*}\leq 0 \end{array}\;i=1,\cdots ,N\;and\;t=\right.1,\cdots ,T\;\varepsilon_{it}\sim N\left(0,\sigma^{2}\right)$$

where *i* represents the 22 provinces of Mongolia, *t* represents different years, *x_it_* is an independent variable, *β* is regression parameters, and $\varepsilon_{it}$ represents the perturbation term. This article applied Stata14 software for panel Tobit regression analysis.

### 2.4. Indicators

This article selected traditional production factors labor and capital as the input indicator and GDP as the output indicator of the economic efficiency in Mongolia. In the existing research of regional eco-efficiency, ecological environment input indicators can include energy, land, water resources, and so on [60,61], and output indicators can include wastewater, SO_2_, NO_2_, PM_2.5_, garbage, and so on. [62,63]. In this article, we selected energy consumption as the ecological environment input indicator and SO_2_ as the ecological environment output indicator. Energy consumption is an important driving force for economic development [64]; it makes a direct link between humans and the natural ecosystem. Energy consumption can indirectly reflect pollution to the natural system caused by greenhouse gas emissions such as carbon dioxide after energy consumption [65]. Therefore, energy consumption has become a core indicator to reflect the pressure of the ecological environment with economic development, especially in background global climate change. The energy consumption in this article is converted to standard coal based on the different types of energy consumption convert coefficients. According to the standard reported by Intergovernmental Panel on Climate Change (IPCC), standard coal can convert to carbon dioxide emission by a coefficient. Therefore, the total energy consumption of standard coal can reflect the CO_2_ emission level, and this article did not include CO_2_ in the undesirable output of the eco-efficiency. SO_2_ is an important pollutant emission from industrial development, and it is an important indicator for comprehensive evaluation of industrial pollution [66]. SO_2_ emissions can indirectly reflect industrial wastewater, solid waste, and other pollution [67]. Moreover, SO_2_ is the main air pollutant continuously monitored in Mongolia. Therefore, in this article, we selected SO_2_ as the undesirable output of Mongolian eco-efficiency (Table 1).

### 2.5. Data

The data in this article were maily from public information sources and are available in Mongolia Statistical Yearbook and Supplementary Materials (Data S1–Data S3). The labor force, GDP, and SO_2_ data in Mongolia were from the Mongolia Statistical Yearbook (2007–2016) [68] with the table of Economically Active Population, Gross Domestic Product (at current prices), and Annual Average Concentration of Pollutants in Air. Due to the fact that economic efficiency and eco-efficiency in this article are calculated each year, GDP and capital investment are both at current prices. The capital in each province is calculated by the following formula:

(6) $$C=\overset{k}{\underset{i=1}{\sum }}GI_{i}\times I_{i}/G_{i}$$

where *C* is capital investment for a province, *G_i_* is the gross production of industry *i*, *I_i_* is capital investment of industry *i*, and *GI_i_* is the gross production of industry *i* in one province. The above data are derived from the Mongolia Statistical Yearbook.

The energy consumption in each province is calculated according to the following formula:

(7) $$E=\overset{k}{\underset{i=1}{\sum }}\overset{l}{\underset{j=1}{\sum }}GI_{i}\times \delta_{ij}EC_{ij}/G_{i}$$

where *E* is energy consumption for a province, *EC_ij_* is the consumption of *j* energy in the *i* industry, *δ_ij_* is the convert coefficient to standard coal of fuel *j* in *i* industry, *G_i_* and *GI_i_* are same as Formula (6). *EC_ij_* is derived from the Mongolia Statistical Yearbook. *δ_ij_* references international standards provided by IPCC [69].

### 2.6. Study Area

This article took Mongolia as an example and estimated the eco-efficiency of 22 provinces (including 21 provinces and 1 capital city), which was divided into 5 regions of Western, Khangai, Cental, Eastern Ulaanbaatar; the 22 provinces are following in Table 2.

## 3. Results

### 3.1. Temporal Evolution of Eco-Efficiency in Mongolia

**Figure 2:**
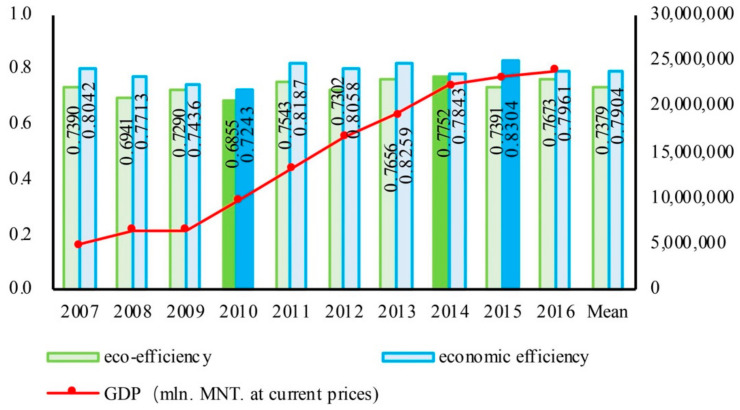
Trend of eco-efficiency and economic efficiency in Mongolia from 2007 to 2016.

As shown in Figure 2, the average value of eco-efficiency in Mongolia from 2007 to 2016 was 0.7379; the highest was 0.7752 and occurred in 2014, the lowest was 0.6855 and occurred in 2010. The average value of economic efficiency in Mongolia from 2007 to 2016 was 0.7904; the highest was 0.8304 and occurred in 2015, and the lowest was 0.7243 and occurred in 2010. The eco-efficiency was slightly lower than economic efficiency, which indicated that the economic development of Mongolia might have an impact on the ecological environment during the study period. From 2007 to 2013, the evolution of eco-efficiency and economic efficiency in Mongolia tended to be consistent, and there was a positive relationship between them. With the slow development of the Mongolian economy from 2007 to 2009, the eco-efficiency and economic efficiency both had a little decline. With steady economic growth from 2010 to 2013, the two efficiencies were stable but did not increase. After 2014, along with the slowdown of growth of the Mongolian economy, eco-efficiency and economic efficiency showed a negative relationship. The basically consistent evolution trend showed that the level of efficiency in economic development had a less negative impact on the ecological environment. Economic efficiency and eco-efficiency had a certain correlation, but the correlation was not clear. Eco-efficiency was a complex dynamic system, which needed to be further analyzed through spatial and temporal dimensions.

### 3.2. Spatial Evolution of Eco-Efficiency in Mongolia

**Figure 3:**
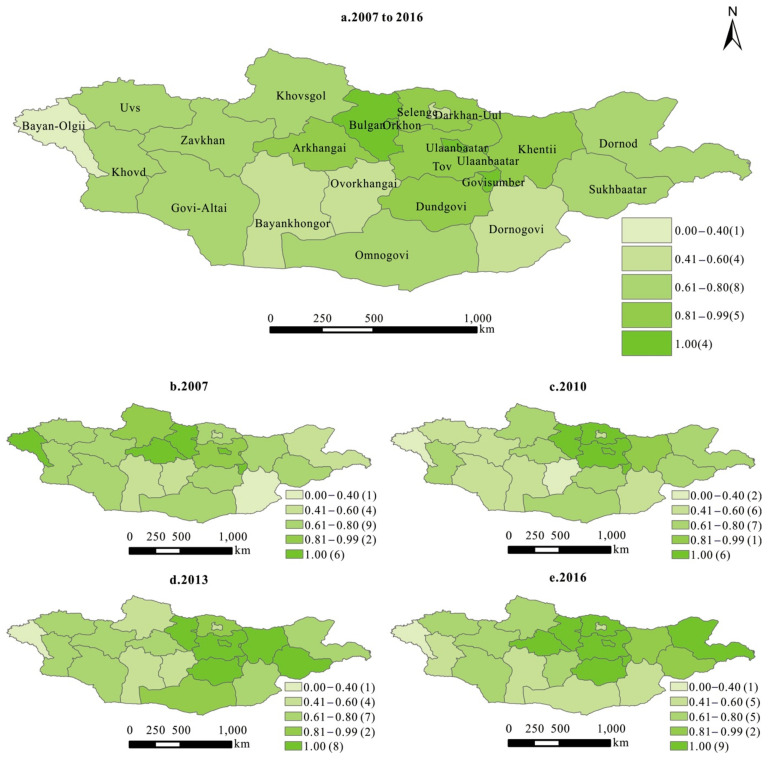
The spatial pattern of eco-efficiency in Mongolia.

During the research period, there were six high eco-efficiency provinces in 2007, including Bayan-Olgii, Arkhangai, Ulaanbaatar, Govisumber, Orkhon, and Bulgan. The high eco-efficiency provinces were increased to eight in 2016, with the addition of Dundgovi, Selenge, Tov, and Dornod (Figure 3). The newly added high-eco-efficiency provinces in 2016 were mainly concentrated in the developed central region of Mongolia and mostly next to the high eco-efficiency provinces in 2007. It was indicated that the spatial pattern of high eco-efficiency provinces showed an agglomeration and spatial spillover effect. Bayan-Olgii in the western Mongolian region is an inefficient province with an undeveloped economy. We found that good location conditions and a high level of economic development lead to a highly eco-efficiency system in the central region of Mongolia. On the other hand, eco-efficiency has been at a low value in the western region of Mongolia due to the constraints of production technology and resource shortages.

**Figure 4:**
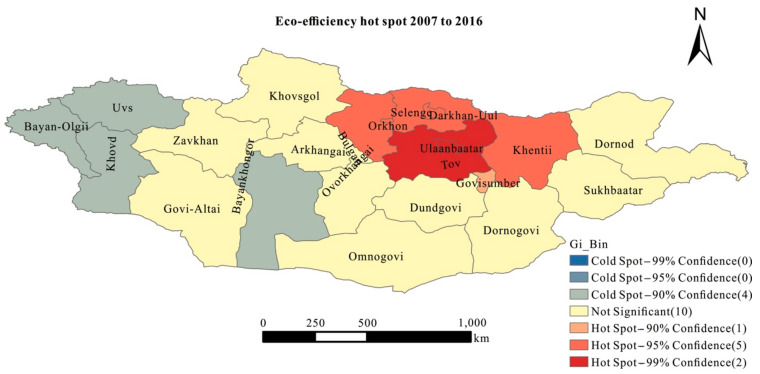
Mongolian eco-efficiency hot spot from 2007 to 2016.

According to the analysis of spatial patterns, the regional eco-efficiency of Mongolia may have a certain spatial correlation. Therefore, this study applied the geographical spatial statistics of the Getis-Ord G_i_* index and the center of gravity analysis to further analyze the evolution characters of eco-efficiency in Mongolia. Based on the Getis-Ord G_i_* index, we analyzed the agglomeration characters of regional eco-efficiency in Mongolia from 2007 to 2016 (Figure 4). The Getis-Ord G_i_* pattern of regional eco-efficiency in Mongolia showed a distribution consistent with the level of economic development, and the pattern difference from Ulaanbaatar to the central region to the western region is obvious. Hot spot provinces were mainly concentrated in Ulaanbaatar and Tov, and the sub hot spot provinces were mainly located in the Selenge River basin near the northern border of Mongolia. Besides Khangai region’s Bayankhongor, the sub-sub cold spot provinces were mainly concentrated in Bayan-Olgii, Khovd, and Uvs, which are located in the western region of Mongolia.

**Figure 5:**
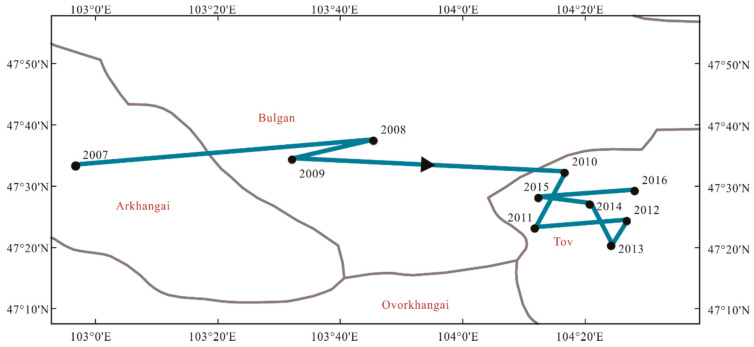
The evolution of Mongolian eco-efficiency center of gravity from 2007 to 2016.

According to Formula (4), the center of gravity of eco-efficiency from 2007 to 2016 in Mongolia can be calculated to analyze the distribution center and its trajectory of eco-efficiency. If it moved in a certain direction, it indicated that the growth of the provinces’ regional eco-efficiencies in this direction were higher than in other provinces. The analysis of the center of gravity of eco-efficiency in Mongolia is significant to finding the law of the elements allocation on eco-efficiency. The results are shown in Figure 5. The gravity centers of eco-efficiency in Mongolia from 2007 to 2016 were in the provinces of Arkhangai, Bulgan, and Tov. The overall mobile range was around 180 km, indicating the eco-efficiency of Mongolia has swung in the past ten years. The trajectory moved from west to east, and the movement direction towards the capital was obvious. After 2010, the speed of eastward movement decreased, and the swing range was about 30 km. From 2010 to 2016, the gap between the eastern region and western region of eco-efficiency in Mongolia became narrowed, and the pattern tended to be more stable. The north–south movement mainly occurred from 2010 to 2016, and moved to the south before 2013, then moved to the north, with a swing range of about 20 km. Therefore, the capital, Ulaanbaatar, impacted the gravity center of eco-efficiency, and it has an obvious spillover effect on eco-efficiency, with a higher input of production technology, labor force, and capital.

### 3.3. Coupling Relationship Analysis of Economic Efficiency and Eco-Efficiency in Mongolia

**Figure 6:**
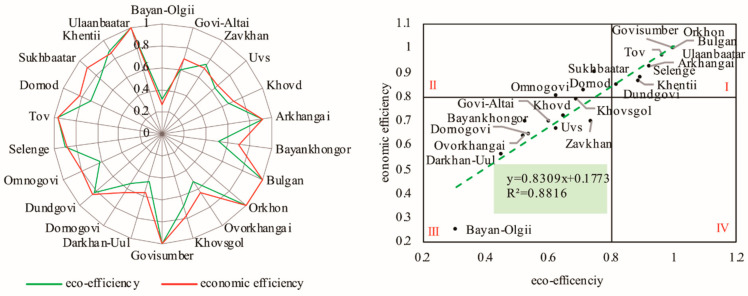
Coupling relationship of eco-efficiency and economic efficiency in Mongolia from 2007 to 2016.

In order to study the coupling relationship between economic efficiency and eco-efficiency in Mongolia, this article analyzed the coupling relationship between economic efficiency and eco-efficiency (Figure 6). The lowest point of economic efficiency and eco-efficiency was found in Bayan-Olgii, and the highest point appeared in Ulaanbaatar, Govisumber, Orkhon, and Bulgan, where the eco-efficiency and economic efficiency were both highest (Figure 6, left). Based on the scatter-point analysis of economic efficiency and eco-efficiency (Figure 6, right), there was an obvious linear positive correlation between eco-efficiency and economic efficiency, and the contribution of unit economic efficiency to eco-efficiency was 0.8309. The higher economic-efficiency provinces can promote higher eco-efficiency. Further, with the high boundary value of the two efficiencies of 0.8, the two efficiencies can be divided into quadrant I of high economic efficiency and high eco-efficiency, quadrant II of high economic efficiency and low eco-efficiency, quadrant III of low economic efficiency and low eco-efficiency, and quadrant IV of low economic efficiency and high eco-efficiency. Mongolia’s 22 provinces were mainly concentrated in quadrants I and III, and some provinces were in quadrant II. Therefore, economic efficiency can improve the eco-efficiency in most provinces in Mongolia; however, the provinces in quadrant II, with high economic efficiency and low eco-efficiency, need to pay more attention to ecologically/environmentally sustainable development.

### 3.4. The Influencing Factors of Eco-Efficiency in Mongolia

In the existing research, economic development [70], green technology [70], industry structure [71], population or labor force [42,72], capital [30,31,72], and environmental regulation [73] were the main influencing factors for improving the regional eco-efficiency or green and sustainable development. Therefore, this article referenced existing research [74,75,76] and incorporated the below indicators in the panel Tobit regression model to analyze the influencing factors of eco-efficiency in Mongolia.

**(1) Economic development.** Economic development is the primary influencing factor of eco-efficiency in existing research [41]. We selected the per capita GDP of the provinces in Mongolia to represent economic development. According to the Kuznets theory of the environment [77], with economic growth, environmental pollution would be aggravated, and when the economy develops to a higher level, it will be improved. In order to discover the relationship between economic development and eco-efficiency, the square of per capita GDP was incorporated into the Tobit model [78].

**(2) Environmental regulation.** Cleff and Rennings [79] defined environmental regulation as the pull–push effect of government policies. Environmental regulations are critical for resource-saving, pollution control, and green development [59]. Existing research has used GDP per capita [59], operating cost of pollution treatment facilities [74], or environmental pollution’s relative intensity [29] as a proxy to environmental regulation. In Mongolia, the priority in waste management during the last decade has been to improve municipal solid waste [7]. Therefore, we used waste facility input as a proxy for environmental regulation.

**(3) Industry structure.** Mongolia’s economic transition has been accompanied by shifting development patterns and changing demographics [9]. The government has already paid more attention to improving the industrial structure. Compared with other industries, the service industry puts less pressure on the ecological environment. A higher proportion of the service sector may be expected to result in better eco-efficiency [59]. Therefore, we used the proportion of the added value of the service industry as a proxy to industrial structure.

**(4) Population density.** Mongolia’s economic development has strongly relied on the population [9]. Higher population density may enhance the labor force and promote economic development, and therefore increase eco-efficiency. Thus, we selected population density represented by unit area people as the influencing factor of eco-efficiency in Mongolia.

**(5) Capital input.** Advanced technologies and green industries’ development need capital support. However, resource consumption and pollution emission might also increase as a result of capital investment. Therefore, we have to find the effect of capital on eco-efficiency in Mongolia to lead to a green financial system. We used per GDP input as a proxy to capital input.

**(6) Green technology.** Green technology can promote the improvement of production technology and reduce the input of resources and energy of unit products [59]. We used per GDP energy input as a proxy to green technology; specifically, a lower per GDP energy input indicates greener technologies.

To avoid the non-stationary problem of parameter estimation caused by different data dimensions, this article selected the natural logarithm of the relevant variables to preserve the characters of panel data (Table 3 and Table 4).

The expression of the panel Tobit model is as follows:

(8) $$\ln EE_{it}=\alpha_{0}+\alpha_{1}\ln GDP_{it}+\alpha_{2}\ln GDP_{it}^{2}+\alpha_{3}\ln EI_{it}+\alpha_{4}IS_{it}+\alpha_{5}\ln PD_{it}+\alpha_{6}RI_{it}+\alpha_{7}RE+\varepsilon_{it}$$

where *i* is 22 provinces of Mongolia, *t* is the research period from 2007–2016, $\alpha_{0}$, $\alpha_{1},\;\ldots \ldots ,\;\alpha_{7}$ are estimation parameters, and $\varepsilon_{it}$ is the random perturbation term. The results of panel Tobit regression are shown in Table 4.

(1) The regression coefficient of per capita GDP and eco-efficiency was negative, and the regression coefficient of the square of per capita GDP and eco-efficiency was positive, so the relationship between per capita GDP and eco-efficiency was a U-shaped curve. This indicates that the eco-efficiency of Mongolia decreased with the growth of per capita GDP nowadays. By the Kuznets theory of the environment, when the per capita GDP reaches a high level, eco-efficiency might increase with the growth of per capita GDP. Regression results showed that per capita GDP increased by 1% and eco-efficiency reduced by 1.67%. This means that Mongolian economic development was still in high energy consumption and high emissions with an unsustainable situation.

(2) The environmental regulation and eco-efficiency did not have a significant negative relationship. In existing research, effective environmental regulation should have a positive effect on eco-efficiency [74]. However, the environmental regulation with waste management in Mongolia did not yet play a positive role, although waste management has improved in recent years, and facilities increased from 809 to 1204 in the period 2007–2016. This means that the effect of pollution control on waste management did not improve the eco-efficiency of Mongolia. It is therefore suggested that, in the future, the policy of energy-saving and emissions reduction should be improved to realize more efficient environmental regulation in Mongolia.

(3) The regression coefficient of population density was 0.091 in a 5% significance test, which indicated that when the population density increases by 1%, the eco-efficiency increases by 0.09%. The positive relationship between population density and eco-efficiency could be due to the high reserve of the labor force, which can improve economic efficiency and increase technical research on ecological/environmental factors. The population distribution in Mongolia was not balanced, and nearly half of the population of the country lived in the capital of Ulaanbaatar during our research period, which supported Ulaanbaatar and its surrounding areas in achieving a high eco-efficiency.

(4) The green technology represented by the per GDP energy input had a negative relationship with eco-efficiency, but a lower per GDP energy input indicates greener technologies. Thus, green technology played a positive role. A decrease of 1% on the per GDP energy input is correlated with an increase of 0.65% in eco-efficiency. Green technology had a great effect on improving the eco-efficiency of Mongolia. This was consistent with Xue’s results of influencing factors of urban eco-efficiency in Western China [32]. Green technology was obviously superior to environmental regulation of waste management in Mongolia during our research period. It is therefore suggested that energy conservation and emission reduction technology should be further explored and used in Mongolia.

(5) The industrial structure did not pass the statistical significance test, but it still had a positive relationship with eco-efficiency. Mongolia is a country rich in mineral resources. The mining industry was a traditional industry with high energy consumption, high emissions, and high pollution. Since 2012, Mongolia has brought three major industries into the national strategic area, including banking and finance, tourism, media, and communications. The service industry has undergone great developments. In Mongolia, the development of traditional mining and the service industry led to the influence of industrial structure on eco-efficiency being statistically insignificant. Therefore, optimizing industrial structure with greening industries suitable for regional sustainable development is an important strategy for Mongolia to improve eco-efficiency and realize green and sustainable development in the future.

(6) The capital input represented by the investment per GDP is associated with eco-efficiency in a negative way but did not pass the significance test. During our research period, more than 80% of foreign direct investment has been invested in the mining field in Mongolia [68]; capital input had difficulty improving eco-efficiency. Therefore, in the future, Mongolia should introduce capital into appropriate strategic greening industries, optimize the investment structure, and make capital play a positive role in optimizing the industrial structure and improving regional eco-efficiency.

## 4. Discussions

From the research results, the eco-efficiency of Mongolia did not increase with economic growth and even had a downward trend. The eco-efficiency was slightly lower than economic efficiency in Mongolia, and there was a strong positive correlation between the two efficiencies. It indicated that Mongolia’s energy consumption and air pollution emissions did not increase much during its economic growth. Mongolia’s industrial structure was dominated by the mining industry, and the high energy consumption and high emissions impacted the ecological environment. This situation was comparable to that of China in the 1990s [80]. Mongolia’s economic growth and its impacts on the ecological environment had not yet been decoupled. This was consistent with the analysis conclusion of urbanization and ecological environment in Mongolia analyzed by Dong et al. (2019) [81]. Since the United Nations SDGs were put forward, Mongolia has taken a series of measures in waste management, industrial greening, and ecological protection, especially land desertification control [7], which were of profound significance to improving eco-efficiency in Mongolia. However, regional eco-efficiency in Mongolia has not significantly increased, indicating that the implementation of the policies has not achieved the coordinated development of the ecological environment and economy. In the future, it is necessary to formulate policies according to the specific conditions of each province based on identifying the influencing factors of economic development, environmental regulation, industrial structure, population quality, and green technology to improve the regional eco-efficiency of Mongolia.

High eco-efficiency provinces in Mongolia were mainly concentrated in the capital, Ulaanbaatar, and its surrounding areas. The distribution of hot spot provinces showed that there were obvious spatial agglomeration and a spillover effect in the high eco-efficiency provinces in Mongolia. Ulaanbaatar and its surrounding areas with high eco-efficiency had favorable productive economic factors, while the western region with low eco-efficiency had a shortage of productive economic factors. Then, the cumulative effect further amplified the spatial differentiation of the economic growth in Mongolia. The Ulaanbaatar region, with better economic productive factors, drives the development of the surrounding provinces through a polarization effect. As the country’s capital, Ulaanbaatar has a prominent central position in economic development, with the agglomeration of universities and scientific research institutes, good infrastructure, and a developed financial market. Employment and educational opportunities were increasingly concentrated in the capital city of Ulaanbaatar [9]. The productive factors of natural resources, capital, and labor force gradually flowed into Ulaanbaatar, promoting the polarization effect to be remarkable. Although energy input and exhaust emissions in Ulaanbaatar were higher than in other parts of the country [82], because of the high level of economic development and the nature of science and technology and the optimized industrial structure, the eco-efficiency of Ulaanbaatar and its surrounding areas was relatively high. Then, due to the spillover effect, the surrounding provinces of Ulaanbaatar achieved high eco-efficiency. The spillover effect and polarization effect aggravated the imbalance of regional eco-economic efficiency and led to the spatial differentiation of the high eco-efficiency and low eco-efficiency provinces in Mongolia. Meanwhile, in the western region of Mongolia, there was a serious shortage of talents, a weak scientific and technological foundation, and a lack of infrastructure for transportation and communication. All these factors severely restricted the human flow, logistics and information flow among provinces, and resisted the spillover effect from the high eco-efficiency provinces. Coupled with the limitation of ecological environment vulnerability, the provinces in the western region fell into a sustainable development dilemma. What is notable is that the government in Mongolia has already focused on agriculture, tourism, industry, energy, and infrastructure as the sustainable development strategic area [13]. In the further, the western region of Mongolia should pay more attention to sustainable development and reference the green development model from Northwestern China, where there are also developing regions with fragile ecological environments [83]. The government of Mongolia can support tourism [84] and new energy industries [85] and implement a grassland ecological compensation policy [86] to achieve sustainable goals on economic growth and ecological environment protection.

The relationship between per capita GDP and eco-efficiency in Mongolia was U-shaped. It was consistent with the findings from Khalid et al. (2014) [33]. However, the relationship between eco-efficiency and GDP growth was still negative. The population density and green technology were important factors to improve Mongolia’s eco-efficiency. To pursue the sustainable development of the economic and ecological environment, Mongolia should accord to the evolution characters of Mongolia’s eco-efficiency and follow the law of development on the economy and ecological environment. In the past decades, Mongolia has taken mining as the strategic industry of the national economic growth. Following Chenery’s industrialization theory [87], the industrial structure centered on the mining industry may exist for a long time in Mongolia. This will restrict Mongolia’s eco-efficiency to improve due to the mining industry consuming a great deal of energy and emitting a significant amount of pollution in the air. Therefore, in order to improve eco-efficiency and achieve green and sustainable development, Mongolia should adopt energy-saving and emission-reduction policies, train relevant technical and theoretical personnel for sustainable development, and use green energy to diversify export towards greener development [88]. Moreover, Mongolia needs to introduce the concept of green finance, taking eco-efficiency as an important indicator to improve the evaluation system of regional green and sustainable development, to realize SDGs of economic growth and affordable and clean energy.

## 5. Conclusions

This article applied SBM to evaluate the economic efficiency and applied Undesira-ble SBM to evaluate the eco-efficiency. Then, this article used Hot Spot and Gravity Center models to analyze the temporal and spatial evolution characters and used panel Tobit Regression to analyze the influencing factors. This article applied the Coupling Relationship (CR) model to analyze the coupling relationship between economic efficiency and eco-efficiency. At last, the conclusions came out as follows:

**(1)**  **Mongolian eco-efficiency development was consistent with economic efficiency; the relationship between eco-efficiency and economic development followed theEnvironmental Kuznets theory**. The eco-efficiency was slightly lower than the economic efficiency, and the efficiency of the economic development is the basis of the high eco-efficiency. The two efficiencies’ range of change was 0.68–0.83, and the increase of eco-efficiency from 2007 to 2016 was not obvious. According to the relationship between eco-efficiency and economic development in Mongolia, it is difficult to achieve high eco-efficiency only through improving economic efficiency or economic growth. A good political system was important to the economic system as well as the ecosystem.
**(2)**  **The spillover effect and polarization effect of economic productive factors have promoted the spatial pattern of Ulaanbaatar and its surrounding areas as the hot spot of eco-efficiency**. Ulaanbaatar, Govisumber, Orkhon, and Bulgan were at the frontier of eco-efficiency. Bayan-Olgii was an inefficient province in the western region. The trajectory of the gravity center of eco-efficiency from 2007 to 2016 obviously moved to the hot spot and high eco-efficiency provinces. The evolution characters of eco-efficiency in Mongolia was a result of the impacts of the spillover effect and polarization effect on the productive factors flowing between the provinces.
**(3)**  **Green technology and industrial structure should pay more attention to improve the eco-efficiency in Mongolia**. Environmental regulation by waste management has not played a positive role in Mongolia’s eco-efficiency. Facing a fragile ecological environment, Mongolia needs to green industries with green technology to reduce energy consumption and pollution emissions, introduce green finance to optimize the industrial structure, and improve the evaluation system with eco-efficiency concepts and measures to realize win–win development of Mongolia’s ecological environment and its economy to achieve SDGs of economic growth and affordable and clean energy.

This study focused on evaluating eco-efficiency in 22 provinces of Mongolia, which is difficult to compare globally and regionally. Further research would carry out cross-regional comparative studies in developing countries, especially those with a fragile ecological environment. The measurement of ecological vulnerability in this study is not enough. Further research should use remote-sensing image data to obtain more ecological indicators, such as Normalized Difference Vegetation Index (NDVI), land degradation, and so on. Future research should integrate ecological vulnerability into the input–output indicators of regional eco-efficiency and comprehensively analyze regional eco-efficiency to achieve more SDGs, including life on land. Future research should establish a better indicator system of influencing factors of regional eco-efficiency in Mongolia, including society, culture, and system, to further discover the driving mechanism of eco-efficiency. Further research should also attempt to deeply explore the temporal and spatial differentiation characters of regional eco-efficiency in Mongolia and apply spatial autocorrelation and geographic detector models to reveal the coordinated mechanisms of sustainable development on the economy and ecological environment in Mongolia.

## Figures and Tables

**Table 1:** Input–output indicators of economic efficiency and eco-efficiency in Mongolia.

Efficiency	Input-Output	Indicator	Data Sources	Unit
Economic efficiency	Input	Labor	Statistical yearbook	Thousand persons
		Capital investment	Formula (6)	bln MNT
	Output	Gdp	Statistical yearbook	bln MNT
Eco-efficiency	Input	Labor	Statistical yearbook	Thousand persons
		Capital investment	Formula (6)	bln MNT
		Energy consumption	Formula (7)	mln ton
	Output	Gdp	Statistical yearbook	bln MNT
	Undesirable output	SO_2_ emissions	Statistical yearbook	Micrograms per m^3^

Note: MNT is the code of Mongolia currency, tögrög.

**Table 2:** Mongolian provinces and territory.

Aimags and the Capital City	Territory (Thousand km^2^)	Aimags and the Capital City	Territory (Thousand km^2^)
**Total**	**1564.1**		
**Western region**	**415.3**	**Central region**	**473.6**
Bayan-Ulgii	45.7	Govisumber	5.5
Govi-Altai	141.4	Darkhan-Uul	3.3
Zavkhan	82.5	Dornogovi	109.5
Uvs	69.6	Dundgovi	74.7
Khovd	76.1	Umnugovi	165.4
**Khangai region**	**384.3**	Selenge	41.2
Arkhangai	55.3	Tuv	74.0
Bayankhongor	116.0	**Eastern region**	**286.2**
Bulgan	48.7	Dornod	123.6
Orkhon	0.8	Sukhbaatar	82.3
Uvurkhangai	62.9	Khentii	80.3
Khuvsgul	100.6	**Ulaanbaatar**	**4.7**

Source: Mongolia Statistical Yearbook 2019 [6].

**Table 3:** Variables selection of driving factors and descriptive statistics.

Variable Meaning	Unit	Variable for Regression	Mean	Std.Dev.	Min.	Max.
Eco-efficiency	-	*EE*	0.738	0.229	0.149	1
Economic development	bln MNT	ln*GDP*	7.926	0.687	6.222	9.504
Economic development	(bln MNT)^2^	ln*GDP^2^*	63.287	10.914	38.713	90.327
Environment regulation	truck	*lnEI*	3.597	0.691	0.693	5.663
Industry structure	%	*IS*	0.325	0.195	−0.329	0.892
Population density	people/km^2^	*lnPD*	0.597	1.700	−1.204	5.725
Capital	bln MNT	*RI*	0.573	0.422	0.136	3.173
Green technology	t/MNT	*RE*	0.182	0.110	0.058	0.644

**Table 4:** Panel Tobit regression analysis of influencing factors of eco-efficiency in Mongolia.

Variable	Coef.	Std.Err.	z	*p* > |z|
lnGDP	−1.674	0.516	−3.24	0.001
lnGDP^2^	0.111	0.033	3.33	0.001
lnEI	−0.044	0.025	−1.8	0.072
IS	0.032	0.064	0.5	0.620
lnPD	0.091	0.037	2.45	0.014
RI	−0.059	0.040	−1.48	0.138
RE	−0.652	0.225	−2.89	0.004
Constant	7.350	1.998	3.68	0.000

Log likelihood = 21.1311.

## Data Availability

The data presented in this study are openly available in Mongolia Statistical Yearbook, it can be find at the website of Mongolia Statistical Information: http://www.1212.mn accessed on 11 October 2021, the reference number is [69].

## Supplementary Materials

The following are available online at https://www.mdpi.com/article/10.3390/ijerph182010719/s1, Data S1: Data for indicators of economic efficiency, Data S2: Data for indicators of eco-efficiency, Data S3: Data for influncing factors of eco-efficiency.
